# Adolescents’ Perspectives on Uptake, Engagement, and Implementation to Inform the Development of a Preventive Transdiagnostic Digital Mental Health Intervention: Qualitative Interview Study

**DOI:** 10.2196/76494

**Published:** 2026-11-02

**Authors:** Anja Hirsig, Noemi Walder, Xenia Anna Häfeli, Stefanie Julia Schmidt

**Affiliations:** 1 Division of Clinical Child and Adolescent Psychology, Institute of Psychology, University of Bern, Fabrikstrasse 8, Bern, 3012, Switzerland, 41 31 684 54 19; 2 Division of Youth Mental Health, Faculty of Psychology, University of Basel, Basel, Switzerland

**Keywords:** adolescent, uptake, engagement, youth mental health, digital, prevention, transdiagnostic

## Abstract

**Background:**

Adolescence is a critical period for mental health prevention; yet, many young people face barriers to help-seeking, including stigma, limited mental health literacy, and restricted access to services. Preventive digital mental health interventions (DMHIs) offer a scalable, low-threshold approach to early support, but uptake and sustained engagement remain limited. Existing research has predominantly relied on quantitative methods and has only partly captured adolescents’ perspectives. Qualitative research is therefore needed to inform the design and implementation of preventive DMHIs.

**Objective:**

This study aimed to explore adolescents’ perspectives on factors influencing the anticipated uptake, expected engagement, and implementation of preventive transdiagnostic DMHIs across person, intervention, and system levels.

**Methods:**

This qualitative study was conducted in Switzerland using a single-interview design in the predesign phase of the development of a transdiagnostic preventive DMHI. Adolescents aged 12‐21 years were recruited through voluntary response sampling via social media and the project website. Semistructured interviews explored experiences with online help-seeking, factors influencing DMHI anticipated uptake and expected engagement, and recommendations for implementation. Interviews were conducted in person or via video call between January and June 2022, audio-recorded, transcribed verbatim, and analyzed using a hybrid deductive-inductive qualitative content analysis. Intercoder agreement was assessed using Cohen κ (κ=0.72).

**Results:**

A total of 48 adolescents (mean age 16.52, SD 2.67 years; 32/48, 72.9% female) participated. In total, 8 main topics and multiple subtopics were identified across person, intervention, system, and cross levels. Person-level factors highlighted the importance of mental health literacy, preuptake communication, and relevance of social networks. Intervention-level factors emphasized usability, personalization, and privacy, while system-level findings reflected expectations for adaptive, integrated, and low-threshold mental health services. Trust and support emerged as cross-level factors for engagement. In addition, participants described several discrepancies between desired intervention characteristics and practical implementation, particularly regarding personalization, anonymity, peer exchange, and human support.

**Conclusions:**

Adolescents’ perspectives highlight that successful implementation of preventive DMHIs requires attention beyond intervention design to preuptake communication strategies, mental health literacy promotion, and integration within adaptive care systems. The tension between adolescents’ desire for immediate human-delivered support and the self-guided nature of preventive DMHIs underscores the need for clear clinical boundaries and explicit escalation pathways.

## Introduction

More than 60% of all mental disorders have their onset before the age of 25 years [1], making adolescence a critical period for mental health promotion and prevention of mental disorders. Despite this need, adolescents often face person-, intervention-, and system-level barriers to accessing professional mental health support [2-5], resulting in low help-seeking rates [6,7] and limited engagement with services [7,8]. This highlights that improving adolescent mental health requires considering the entire pathway from help-seeking to accessing and engaging with appropriate services [9].

Digital mental health interventions (DMHIs) offer a low-threshold option for seeking, accessing, and receiving mental health support [10]. Further advantages of DMHIs include their frequent availability at no cost, scalability, and accessibility, making them a viable option for hard-to-reach populations [8,11]. A growing number of studies supports the efficacy of DMHIs in both promoting mental health and preventing specific mental disorders in adolescents [12-14]. However, some traditional barriers, such as fear of stigma or low mental health literacy, persist [11,15,16]. Additionally, DMHIs present their own challenges, related to technology, privacy and data protection, and design, which may limit their uptake and engagement [15,17,18].

In this study, “uptake” is defined as initial access to or first use of a preventive DMHI, while “engagement” refers to any subsequent use of the intervention beyond the initial access, including frequency and duration of use [19-21]. The capability-opportunity-motivation behavior (COM-B) model provides a useful framework for understanding how interventions can enhance uptake and engagement [22]; its application in this study is described in the Methods section. A systematic review [16] identified a range of person-specific factors to be relevant for the engagement of young people with DMHIs, categorized by capability (eg, necessary knowledge and skills), motivation (eg, perceived usefulness and helpfulness), and opportunity (eg, trust and anonymity). In addition, intervention-specific factors included acceptable, usable, and contextually suitable features that fit adolescents’ needs and daily routines [16]. Notably, the studies included in this review did not distinguish between uptake and engagement or used the terms interchangeably [16], thereby impeding the interpretation and implementation of the results. Thus, further research is needed to better understand the most relevant factors influencing uptake and engagement [15,19]. While person- and intervention-level factors have been widely explored, system-level factors—including how DMHIs could be integrated into existing mental health care structures—remain comparatively understudied. Therefore, further research is needed to comprehensively investigate person-, intervention-, and system-level factors shaping adolescents’ uptake and engagement with DMHIs [23]. However, most previous studies have used quantitative surveys with predefined response options, limiting opportunities for adolescents to express their own perspectives [24]. In contrast, qualitative or mixed method approaches can provide a more in-depth and personalized exploration of factors influencing adolescents’ uptake and engagement with DMHIs [25].

A participatory, co-design approach is increasingly regarded as best practice for developing youth mental health interventions because it actively involves adolescents from conceptualization and development to the evaluation and implementation of DMHIs [26,27]. Co-designed DMHIs offer many benefits, such as being more relevant to youth, improving usability, fostering trustworthiness, and facilitating real-world implementation [28-30]. To date, adolescents’ perspectives have primarily been considered in the development and evaluation of DMHIs targeting specific mental health problems, such as anxiety or depression [31,32]. However, despite the potential benefits of co-design, its application in the development of preventive DMHIs remains limited [33]. Given the dynamic and transdiagnostic nature of emerging psychopathology [34,35], co-designed transdiagnostic DMHIs hold significant promise for prevention as these interventions address a broad range of mental health problems and may enhance both engagement and overall mental health outcomes [36,37]. While some transdiagnostic preventive DMHIs have been co-designed with adolescents [38], they have primarily focused on developing and evaluating prototype interventions. In contrast, less attention has been given to involving adolescents in the predesign or discovery phase, that is, the stage that precedes the generative design process and focuses on identifying the needs and preferences of the target group [31,39]. The predesign phase seems particularly critical because it establishes the foundational understanding that guides subsequent design decisions. Without a thorough understanding of adolescents’ needs, preferences, and barriers from the outset, later design efforts risk producing interventions that are misaligned with the target group’s lived experiences and priorities [40,41].

This study (BePart study) addresses this gap as part of a larger co-design project (ie, EMPATIA study, [36]) to develop and evaluate a preventive transdiagnostic DMHI for adolescents, following the co-design frameworks described by Bevan Jones et al [31] and Sanders and Stappers [39]. This paper reports on the predesign phase, in which qualitative interviews with adolescents were conducted to explore their perspectives on factors influencing anticipated uptake and expected engagement with preventive transdiagnostic DMHI. Against this background, this study aimed to explore adolescents’ perspectives on anticipated uptake, expected engagement, and implementation to inform the development of a preventive transdiagnostic DMHI through answering the following research questions. First, at the person and intervention level, what factors are relevant for adolescents to enhance uptake and engagement with preventive transdiagnostic DMHIs? Second, at the system level, what needs regarding preventive transdiagnostic DMHIs and the mental health care system in general are voiced by adolescents to enhance uptake and engagement? Third, what factors are relevant to enhance uptake and engagement with preventive transdiagnostic DMHIs across levels?

## Methods

The following parts of the manuscript are reported according to the Consolidated Criteria for Reporting Qualitative Research (COREQ) [42].

### Study Design

As part of the EMPATIA project [36], the BePart study constituted the predesign phase of an iterative co-design process to develop a preventive transdiagnostic DMHI for adolescents. Through semistructured interviews, adolescents’ needs, preferences, and perceived barriers were explored to inform the development of a prototype, which was subsequently evaluated and refined through focus group interviews with adolescents during the design phase. The developed intervention was then evaluated in a randomized controlled trial as part of the broader EMPATIA project. The BePart study was conducted in German-speaking cantons of Switzerland. Between January and June 2022, adolescents from the general population were recruited to participate in one semistructured interview.

### Ethical Considerations

This study was approved by the Ethics Commission of the Faculty of Human Sciences of the University of Bern (2021-12-00003) and is in accordance with Articles 3(k) and 23 of the Swiss Human Research Act (HFG), which permit capable adolescents aged ≥14 years to provide their own written informed consent for minimal-risk research. Upon contacting the study team, potential participants received the study information and informed consent documents by mail. Participants aged ≥14 years signed the informed written consent form themselves and returned it via mail. For participants aged 12 or 13 years, written informed consent was obtained from a parent or legal guardian, and written assent was obtained from the participant. Participation was voluntary, and participants were informed that they could withdraw at any time without consequences.

Interview data were pseudonymized. Prior to the interview, each participant generated a personal code. Audio recordings were stored under this code. At no point during the interview was the participant’s name recorded. The code key linking participants’ identities to their pseudonyms was stored separately and was accessible only to the principal investigator. Audio recordings were deleted following transcription. All data were stored in a password-protected folder accessible only to the principal investigator. Only deidentified data were used for analysis and reporting. Participants received CHF 20 (∼US $21.2, as of January to June 2022) in the form of a voucher after the interview. Additionally, they were reimbursed for potential traveling expenses.

### Participant Selection

Participants were recruited through voluntary response sampling. Study information was distributed online (via social media profiles and the project website), through flyers, media interviews, and cooperation partners. Interested participants were asked to contact the study team via email, direct messages on social media, or phone call to arrange a date for the interview. To be included, participants had to (1) be between 12 and 21 years old, (2) have their main residency in Switzerland, and (3) understand and speak German. Prior personal experience with DMHIs and/or mental health problems did not serve as an inclusion or exclusion criterion. Although participants ranged from 12 to 21 years, we use the term adolescents throughout the paper in line with contemporary developmental definitions that conceptualize adolescence as an extended developmental period [43,44].

### Study Procedures

After having provided consent, participants and interviewers agreed on the study information via mail. Participants (or their legal guardians when aged <14 years) needed to sign the informed consent form and to return it via mail. Participants and interviewers then agreed on a time and date for the interview, either in person or via online video call. Prior to the interview, all participants were asked to complete an anonymous online survey about help-seeking and psychological distress (refer to Materials section). Only the interviewer and the participant were present at the date of the interview. The interview started with demographic questions about age, gender identity (female, male, and other), and sociodemographic data. Race and ethnicity were not collected because the study was not designed to examine differences in intervention perceptions or engagement across demographic groups. Thereafter, participants were again informed on the study procedures in a standardized manner (Multimedia Appendix 1). If the participants had no further questions, the interviewer started the audio recording and carried out the interview.

Given the sensitive nature of the interview topics, a participant-centered approach emphasizing participant safety and autonomy was applied, consistent with recommendations for qualitative research on such topics [45-47]. All interviewers were advanced master’s students specializing in clinical child and adolescent psychology with prior experience conducting clinical interviews. Before data collection, they completed a 2-day training workshop covering communication and active listening skills, recognizing signs of distress, maintaining appropriate boundaries, and procedures to support participant safety and autonomy. A predefined procedure for managing participant distress was established. If participants showed signs of distress, interviewers paused the interview, assessed the participant’s immediate well-being, and offered the option to continue, take a break, skip questions, or terminate the interview. Participants were informed that participation was voluntary and that they could pause or withdraw at any time without consequences. Participants requiring further support were provided with written information on available support services. If additional clinical support was required, interviewers could consult SJS, a licensed psychotherapist, during or after the interview, and participants could be referred to the outpatient service of the involved clinical division. Established clinical procedures for acute safety concerns, including urgent referral when clinically indicated, were available. Potential challenges and critical situations were discussed during regular team supervision meetings.

The research team regularly reviewed the latest interview audios to monitor data saturation, assessing whether any new key topics emerged. This process continued until the team agreed that no new information leading to possible codes for answering the research questions had emerged, indicating that recruitment could be concluded.

### Personal Characteristics

The semistructured interviews were conducted by 4 female master’s students in their last term (Christina Hiltbrunner, Nika Saxer, Taina Thees, and Vera Bächler). The interviewers did not have previous experience in qualitative interviewing but were experienced in conducting clinical diagnostic interviews with adolescents. The interviews were coded by NW, PhD, and AH, MSc. The coders and interviewers were supervised by SJS, a professor in Clinical Psychology and licensed psychotherapist for children and adolescents.

Prior to data collection, interviewers conducted pilot interviews with adolescents that were reviewed within the research team to refine interview questions and ensure comprehensibility. These pilot interviews were complemented by structured role-plays among the interviewers, which were discussed to develop a shared understanding of appropriate open-ended follow-up questioning and to identify potential interviewer biases. Throughout data collection, interviewers participated in weekly team supervision meetings with SJS, during which emerging challenges were discussed. These procedures were implemented to enhance the credibility and trustworthiness of the data, consistent with established quality criteria for qualitative research [48,49].

### Materials

The online survey included the General Help Seeking Questionnaire (GHSQ) [50], the Actual Help Seeking Questionnaire (AHSQ) [51], and the Kessler Psychological Distress Scale (K10) [52]. The semistructured interview guide was developed specifically for this study (Multimedia Appendix 1). It was informed by factors influencing help-seeking [2], the COM-B model [22], and by the intervention-level factors associated with engagement in DMHIs identified in the systematic review by Liverpool et al [16]. Accordingly, the interview questions covered the entire pathway from help-seeking to the uptake and continued engagement with a proposed preventive DMHI. The interview guide was reviewed by an external qualitative researcher who was not otherwise involved in the study. Based on this feedback, several questions were revised to place greater emphasis on participants’ behaviors and experiences rather than opinions. Additionally, the interview questions were subsequently piloted with 2 adolescents to assess comprehensibility and estimate the interview duration. The pilot interviews were not included in the final dataset.

The interview began by exploring participants’ general use of digital applications and online help-seeking behaviors (eg, “Where would you look online for information about mental health problems and for help if needed?”). Participants were then introduced to transdiagnostic, preventive DMHIs to establish a shared understanding of the intervention type before discussing factors that would influence their willingness to start using and continue engaging with such interventions (eg, “What would you need to tell your friends about the DMHI to get them to start using and engaging with it?”). Participants subsequently evaluated 21 predefined intervention features [16] (eg, video content or text) by rating their importance on a scale ranging from 1 (“not important at all”) to 10 (“very important”). The ratings served solely as structured prompts to facilitate discussion. Participants were explicitly asked to elaborate on their responses, suggest implementation strategies, introduce additional features, or reject items they considered unimportant or irrelevant. The predefined features were drawn from the systematic review by Liverpool et al [16] and reflect their 3 intervention-specific dimensions of acceptability, usability, and suitability. Within the acceptability dimension, predefined features included content format options (information texts, animated videos, videos with real-life adolescents or influencers, and everyday-life tasks), exchange options (with peers, e-coaches, and professionals), and the inclusion of parents. Within the usability dimension, features included layout and design, offline availability, and the self-administered format of the program. Within the suitability dimension, features included options to adapt content (eg, additional examples, own profile, and choice of avatar), data security and anonymity settings, and feedback mechanisms (specific progress feedback or integrated progress bar). This study thus operationalizes intervention-specific dimensions by Liverpool et al [16] at a more granular level, drawing directly on adolescents’ own perspectives in a preventive transdiagnostic context. The interview concluded with questions on personalization (eg, “How could we best tailor the DMHI to each individual participant?”). All interview data including all follow-up calls were used in the qualitative analysis.

### Data Analysis

All interviews were transcribed verbatim following the transcription rules described by Kuckartz [53]. Data were analyzed using structured qualitative content analysis [54] in MAXQDA [55]. This approach aims to identify and systematize topics and subtopics and examine relationships between categories through an iterative process comprising the following seven phases: (1) initial work with the text, (2) development of main topics, (3) coding data with main topics (first coding cycle), (4) inductive development of subtopics, (5) coding data with subtopics (second coding cycle), (6) simple and complex analyses, and (7) writing up of results. Research questions can continuously be adapted up to phase 6 [54]. In the present analysis, the originally planned research questions distinguished between personal, intervention, and system-level factors. During the analysis, it became apparent that factors relevant to the uptake and engagement with preventive transdiagnostic DMHIs could not always be meaningfully assigned to a single level. Therefore, the third research question was refined to explicitly capture factors operating across levels.

A hybrid deductive-inductive approach was applied, consistent with theory-informed qualitative content analysis [54]. The initial coding framework was developed deductively based on the COM-B model [22] and a systematic review on factors influencing engagement with DMHIs among young people [16]. At the person level, initial topics represented the COM-B constructs capability, opportunity, and motivation. At the intervention level, topics were based on intervention-related factors identified in previous DMHI research [16], namely acceptability, usability, and suitability. In addition, a broad help-seeking topic was included [2] to reflect the structure of the interview guide, which examined adolescents’ help-seeking pathway from awareness of mental health needs to considering, accessing, and engaging with preventive DMHIs. These topics guided the first coding cycle, which was conducted independently by both coders, NW and AH, using a subset of 4 interviews.

During subsequent coding cycles, the framework was refined iteratively based on the interview material. Subtopics were developed inductively, allowing participants’ perspectives and unexpected concepts to shape the final analytical structure. Participants frequently described expectations extending beyond a single intervention toward broader visions of preventive mental health care; therefore, system-level factors were added as an inductively derived main topic. Similarly, discrepancies between adolescents’ expectations and perceived implementation possibilities emerged as a cross-cutting topic. The distinction between anticipated uptake and expected engagement also became increasingly relevant during analysis, as perspectives on DMHI characteristics differed across stages of the help-seeking process. The “access to health care” framework by Levesque et al [56], adapted for young people by Werlen et al [9], was consulted to support interpretation of this process dimension. Additionally, the health belief model [57] was used to refine terminology for inductively developed belief-related subtopics. These frameworks supported interpretation but were not used as predefined coding categories. Table 1 provides an overview of the deductive theoretical starting points to the inductively developed final topics, indicating the theoretical frameworks and interview questions associated with each, and providing commentary on the analytic decisions made. After establishing the coding system, the coders independently coded 2 interviews to assess intercoder agreement. Agreement was evaluated using MAXQDA [55] by comparing all coded segments on a document level, resulting in 73.4% agreement and a Cohen κ of 0.72. NW and AH subsequently discussed all coding discrepancies, including differences in assigned codes and uncoded segments. Most differences occurred in the subtopic coding of Beliefs about DMHIs influencing anticipated uptake and engagement. Therefore, corresponding code definitions were refined to improve distinction between subtopics. Following this discussion, full agreement was reached, and the revised coding system was applied to the remaining interviews, with NW and AH independently coding half of the interviews each. Weekly meetings were held throughout the coding process to ensure consistency. After completion of coding, AH reviewed all coded segments, and remaining uncertainties were resolved through discussion. Representative participant quotations were selected from the original German transcripts and translated into English by the first author (AH) and additionally reviewed by the senior author (SJS), both native German speakers proficient in English. The translations aimed to preserve the conceptual meaning and participants’ intended message rather than providing a literal word-for-word translation.

The feature ratings were analyzed quantitatively and reported separately in Multimedia Appendix 2. They were not incorporated into the qualitative coding or theme development.

**Table 1.** Development from deductive starting topics to inductively derived final topics^a^.

Deductive starting topics	Interview question	Inductively derived final topics	Comment
Help seeking [2]	Q1-3a-c	Facilitating factors in the awareness phase of help seeking [2,9,56]	The 2 final personal-level topics were developed inductively through iterative analysis of the interview data. The initial topics (help seeking, capability, opportunity, and motivation) served as an analytical starting point of departure, while the final topics were refined to represent recurring patterns identified across participants’ accounts.
Capability [16,22], opportunity [16,22], and motivation [16,22]	Q3d, 4‐8	Beliefs about DMHIs^b^ influencing anticipated uptake and engagement [57]	The 2 final personal-level topics were developed inductively through iterative analysis of the interview data. The initial topics (help seeking, capability, opportunity, and motivation) served as an analytical starting point of departure, while the final topics were refined to represent recurring patterns identified across participants’ accounts.
Suitability [16]	Q3e,9‐12	Requirements for DMHI features [16] Preferences regarding presentation of DMHI content [16]	The two final intervention-level topics were developed inductively through iterative coding and constant comparison of the interview data. The initial topics (suitability, usability, and acceptability) informed the analytical process, whereas the final topics were refined to capture participants’ perspectives and experiences.
Usability [16]	Q3e,9‐12	Ideas and wishes for adaptive, integrated, and low-threshold mental health services [3,58-60]	The final system-level topic emerged inductively after extensive engagement with the interview transcripts. Relevant system-level frameworks were consulted only post hoc to contextualize the findings, not to derive the category.
Acceptability [16]	Q3e,9‐12	Trust [16] Support offered through a DMHI [16] Discrepancies between expectations and implementation possibilities	The 3 final cross-level topics emerged inductively during iterative analysis of the interview data as recurring cross-cutting themes.

a The deductive-inductive hybrid qualitative content analysis started with 7 deductively derived topics and resulted in 8 inductively developed final main topics.

b DMHIs: digital mental health interventions.

### Author Reflexivity

The first author (AH) was actively engaged in developing a transdiagnostic preventive DMHI at the time of data collection, and NW brought in expertise from prior research on a similar DMHI for adolescents with social anxiety. Both initially framed topics within existing theoretical models, which they actively challenged through reflexive diary-keeping and regular team discussions to remain open to new insights. Additionally, 4 master’s students conducted the interviews. Their closer age to participants may have facilitated rapport and open discussion, while also increasing the possibility that shared assumptions about digital engagement were left unexplored. Further reflexivity considerations, including the influence of the all-female team composition and the time lag between data collection and analysis, are detailed in Multimedia Appendix 3.

## Results

### Overview

A total of 48 participants were interviewed (6 in person, 42 via online video call). Interviews lasted between 30 and 85 minutes (mean 50.04, SD 11.58 min). Thirty of 48 participants were recontacted via telephone to elaborate on previous responses such as provided feature ratings, with these follow-up conversations lasting between 1 and 28 minutes (mean 12.28, SD 7.95 min). Moreover, 4 participants had a personal connection with one of the interviewers. To minimize potential bias, they were interviewed by an interviewer with whom they had not previously interacted.

The sample consisted of predominantly well-educated, female participants aged 12 to 21 years (refer to Table 2 for the sample description). During the interviews, 9 (18.8%) participants reported prior use of a digital application related to (mental) health (eg, information websites, chats, and meditation apps).

**Table 2.** Sample characteristics (n=48).

Variables	Values
Age (years), mean (SD)	16.52 (2.67)
Gender, n (%)	
Female	35 (72.9)
Male	12 (25.0)
Nonbinary	1 (2.1)
Education level^a^, n (%)	
University	4 (8.3)
Higher secondary school	28 (58.3)
Apprenticeship	2 (4.2)
Lower secondary school	9 (18.8%
Primary school	5 (10.4)
Psychological distress (K10^b^; n=47), mean (SD)	21.83 (6.59)
General help seeking (GHSQ^c^; n=46), mean (SD)	3.16 (0.79)
Friend (nonrelative)	5.37 (1.79)
Parent	4.57 (1.95)
Internet	4.52 (1.91)
Intimate partner	3.85 (2.52)
Other relative	2.98 (1.87)
School psychologist or social worker^d^	2.33 (1.38)
(Child and adolescent) Psychotherapist^d^	3.02 (1.68)
(Child and adolescent) Psychiatrist^d^	2.96 (1.79)
Counselor^d^	2.59 (1.60)
Doctor/GP^d,e^	2.00 (1.25)
Teacher^d^	2.24 (1.58)
Telephone helpline^d^	2.63 (1.60)
Online peer chat^d^	2.09 (1.58)
Online chat with a professional^d^	3.02 (1.73)
Online self-help program^d^	3.24 (1.82)
Would not seek help	2.98 (1.98)
Actual help seeking (AHSQ^f^); lifetime (n=46), n (%)	32 (69.6)
No help	14 (30.4)
Formal help	19 (41.3)
Informal help	30 (65.2)
Actual help seeking (AHSQ); past 12 months (n=46), n (%)	32 (69.6)
No help	14 (30.4)
Formal help (n=32)	11 (23.9)
Informal help (n=32)	29 (63)

a Education level refers to current level of education.

b K10: Kessler Psychological Distress Scale.

c GHSQ: General Help-Seeking Questionnaire.

d Formal sources of help: Actual Help-Seeking Questionnaire [51], General Help-Seeking Questionnaire [50], and Kessler Psychological Distress Scale [52].

e GP: general practitioner.

f AHSQ: Actual Help-Seeking Questionnaire.

### Content Analysis

#### Overview of Content Analysis

The deductive-inductive analysis approach identified 8 main topics reflecting the entire process from help-seeking to anticipated uptake and sustained expected engagement with preventive DMHIs. Of the total, 5 of these topics represented factors primarily operating at the person-, intervention-, or system-level, while three cross-level themes reflected concepts that extended across analytical levels: (1) trust; (2) support, reassurance, and connection; and (3) discrepancies between adolescents’ expectations and implementation possibilities. Table 3 provides a comprehensive overview of all final main topics and subtopics across analysis levels; the detailed codebook is provided in Multimedia Appendix 4.

**Table 3.** Main topics and subtopics by analytical level.

Level	Main topic	Subtopics
Person	Facilitating factors in the awareness phase of help seeking	Basic knowledge about DMHIs^a^ and their relevance for prevention Own previous experiences with digital (mental) health services Improving knowledge about mental health Improving or maintaining personal mental health
Person	Beliefs about DMHIs influencing anticipated uptake and engagement	Providing entertainment and feedback Beliefs about general helpfulness Critical beliefs about DMHIs and their usefulness for prevention Expected stigmatization related to uptake and engagement of DMHIs
Intervention	Requirements for DMHI features	Free of charge State-of-the-art app
Intervention	Preferences regarding presentation of DMHI content	Content is easy to understand and follow Variety in delivery format Inclusivity in content Length and amount of content Personalization Individualization
System	Ideas and wishes for adaptive, integrated, low-threshold mental health services	Adaptive in intensity Integrated and accessible Low-threshold and continuous support
Cross-level	Trust	Recommendations from trusted sources Trustworthy provider Testimonial reports Options for anonymous use
Cross-level	Support, reassurance, and connection	Help is available and real (ie, help being genuinely accessible) Others share similar experiences Wanting to feel seen, understood, and taken seriously
Cross-level	Discrepancies between expectations and implementation possibilities	Personalization without personal information Permanent support with low personal engagement Self-disclosure by others but not by themselves

a DMHIs: digital mental health interventions.

#### Person Level

##### Facilitating Factors in the Awareness Phase of Help-Seeking

Three key facilitating factors shaped adolescents’ initial awareness of and willingness to consider using a preventive DMHI: (1) basic knowledge about mental health, DMHIs, and their preventive role; (2) recommendations from trusted sources; and (3) previous experiences with digital mental health services. These factors correspond to the COM-B constructs of capability (knowledge and symptom recognition) and opportunity (social influences and recommendations) [22]. According to the access to health care framework [9,56], they relate to the awareness phase, during which individuals recognize a need for support and become aware of available services.

A noteworthy finding was that participants described specific informational prerequisites that extended the COM-B construct of capability beyond general mental health literacy. Rather than merely knowing that a DMHI existed, they wanted concrete information about its goals, provider, time commitment, and registration process before considering its uptake. This finding suggests that uptake barriers begin before any contact with the intervention itself, highlighting the importance of providing this information during preuptake communication. Participants described several trusted sources of information, including schools, peers, and family members.

*Well, in my school there are posters everywhere promoting [...] some website or an app. I would probably try these first [if I ever needed help for my mental health]*.

[TN21, 17 years, female]

Interestingly, recommendations from peers and family members emerged as the most influential source of awareness, suggesting that adolescents’ social networks may play a greater role in promoting awareness of and facilitating uptake of DMHIs than professional or institutional channels. Rather than simply influencing satisfaction with a single intervention, participants described early experiences with digital mental health services as shaping their general openness toward future DMHI use, which influenced their uptake intention in opposite directions. Negative early experiences, such as receiving unhelpful advice or encountering long waiting times, created lasting barriers to future online help-seeking. In contrast, positive experiences increased participants’ willingness to return to digital support and to explore new tools during periods of distress.

##### Beliefs About DMHIs Influencing Anticipated Uptake and Engagement

Beliefs about the potential influence of DMHIs on uptake and engagement varied considerably across participants. Perceived benefits included improving mental health literacy, maintaining or enhancing psychological well-being, and providing engaging features such as feedback or gamification. These beliefs primarily reflected the COM-B domain of reflective motivation [22], as participants evaluated whether a DMHI would be personally valuable and worth using. They also corresponded with the health belief model’s emphasis on perceived benefits and barriers as determinants of health-related behavior [57]. Across participants, however, one overarching belief consistently emerged: uptake and sustained engagement were expected to depend on whether the intervention would be perceived as personally helpful. Yet, when participants were asked to define what “being helpful” meant, their responses varied substantially, and no shared definition emerged. Thus, helpfulness appeared not to represent a fixed expected characteristic of a DMHI, but rather a subjective criterion through which adolescents assessed its perceived value.

*Well, if it would personally help me, then [I would] definitely [use it] in those moments when I’m not feeling well, like when my mental health isn’t good or whatever. And if I know, it helps then I would definitely use it. Or maybe also as a preventive measure, maybe to avoid feeling mentally unwell in the future or build some kind of protection against that*.

[TN16, 15 years, female]

For several participants, positive expectations regarding DMHIs coexisted with doubts about whether digital interventions could adequately address their individual needs. These critical beliefs emerged as inductively derived subtopics and included a preference for managing mental health problems independently or seeking face-to-face support, uncertainty about the effectiveness of digital interventions, concerns that the content would not be applicable to their personal circumstances, and the belief that professional support should only be sought for more severe mental health problems.

*I am more inclined to think that it’s of little use to me, but anyway, of course I don’t rule it out completely and say “no, I won’t even try it, it’s no use anyway” because maybe there will finally be a program that actually delivers results*.

[TN33, 19 years, male]

Another recurring belief concerned anticipated stigma. Many participants distinguished between being willing to use a DMHI and being willing to be seen using one. Having a DMHI visibly installed on their device or being observed engaging with it was associated with discomfort, embarrassment, and fear of being judged by others. Within the COM-B framework, anticipated stigma represents a barrier related to social opportunity, as concerns about being observed or judged by others may restrict adolescents’ willingness to initiate DMHI use, even when they perceive potential personal benefits. Therefore, concerns regarding privacy, discretion, and anonymity appeared to be relevant for adolescents’ willingness to engage with preventive DMHIs and were closely linked to the anonymity theme discussed further in this study.

### Intervention Level

#### Overview

Intervention-level topics included participants’ evaluations of predefined intervention features (Multimedia Appendix 2) and an additional theme that emerged inductively, namely expectations regarding inclusive intervention content.

#### Requirements for DMHI Features

Key features of DMHIs considered essential “must-haves” included the DMHI being free of charge and being “state-of-the-art,” that is, being perceived as a high-quality, well-designed, and smartphone-friendly app. Interestingly, rather than being perceived as features that actively enhance expected engagement, these characteristics represented minimum expectations that influenced whether adolescents would consider downloading or trying the intervention in the first place. Consistent with the results of the systematic review on intervention-level factors in DMHIs [16], these findings illustrate how specific intervention components shape users’ initial perceptions before any active willingness to engage with the intervention occurs. In particular, participants highlighted the importance of a strong first impression and appealing design for initial acceptability, noting that they would be unlikely to download the app otherwise. Additionally, an intuitive user experience, particularly regarding ease of navigation and interaction, was expected to be crucial for sustained engagement, reflecting the role of usability in supporting continued use. For example, one participant stated:

*I would use it on my phone because I always have it with me. And if it’s a bit annoying to use it on there, you know, like to navigate or if everything keeps shifting around and is just kind of awkward, I think with time it would start to really frustrate me*.

[TN35, 19 years, male]

These features were therefore regarded as basic quality standards rather than benefits that actively enhanced engagement.

#### Preferences Regarding Presentation of DMHI Content

Participants consistently described intervention content that minimized cognitive burden as a perceived facilitator of sustained engagement. Clearly structured content, concise text, short videos, and simple exercises with minimal technical jargon were perceived as making it easier to engage with a DMHI. Conversely, content that appeared lengthy or overwhelming was expected to discourage engagement. These findings align with the usability dimension of the framework, highlighting the importance of reducing effort required to engage with the intervention [16]. Participants further emphasized the importance of presenting content in different formats to accommodate individual preferences, different learning styles, and maintain interest over time. An additional finding that emerged inductively was that participants did not support gender-based content differentiation. Instead, they favored the idea of inclusive, gender-neutral and nonstereotypical representation across examples, avatars, and language. Consequently, they rejected the idea of having separate intervention versions based on gender. Instead, they favored the possibility of adaptations to the level of difficulty rather than changes to the core content. These preferences suggest that acceptability is enhanced when users perceive the intervention as broadly relevant and inclusive without reinforcing stereotypes.

A recurring expectation concerned tailoring of the intervention, consistent with intervention-level frameworks [16] that identified tailoring as a mechanism for increasing perceived relevance while preserving user autonomy. Participants distinguished between individualization, whereby users actively select intervention features, and personalization, in which content is automatically adapted based on user characteristics, input or interactions. Both approaches were expected to increase the perceived relevance of the intervention and thereby facilitate sustained engagement. Suggestions for individualization included customizable design elements, privacy settings, interaction options, and content selection. For personalization, participants proposed an initial (or recurring) questionnaire to tailor content according to users’ responses and self-identified challenges.

*I imagine that in the beginning, you fill out a questionnaire to assess where there are challenges. This could provide some guidance on what to focus on, while still allowing users to input their own areas for improvement. I would suggest that the program be somewhat personalized from the start*.

[TN33, 19 y, male]

Throughout the interviews, participants stated specific ideas on how to implement certain features in the DMHI to enhance anticipated uptake and/or expected engagement (refer to Figure 1 and Multimedia Appendix 5 for a detailed description).

**Figure 1.**
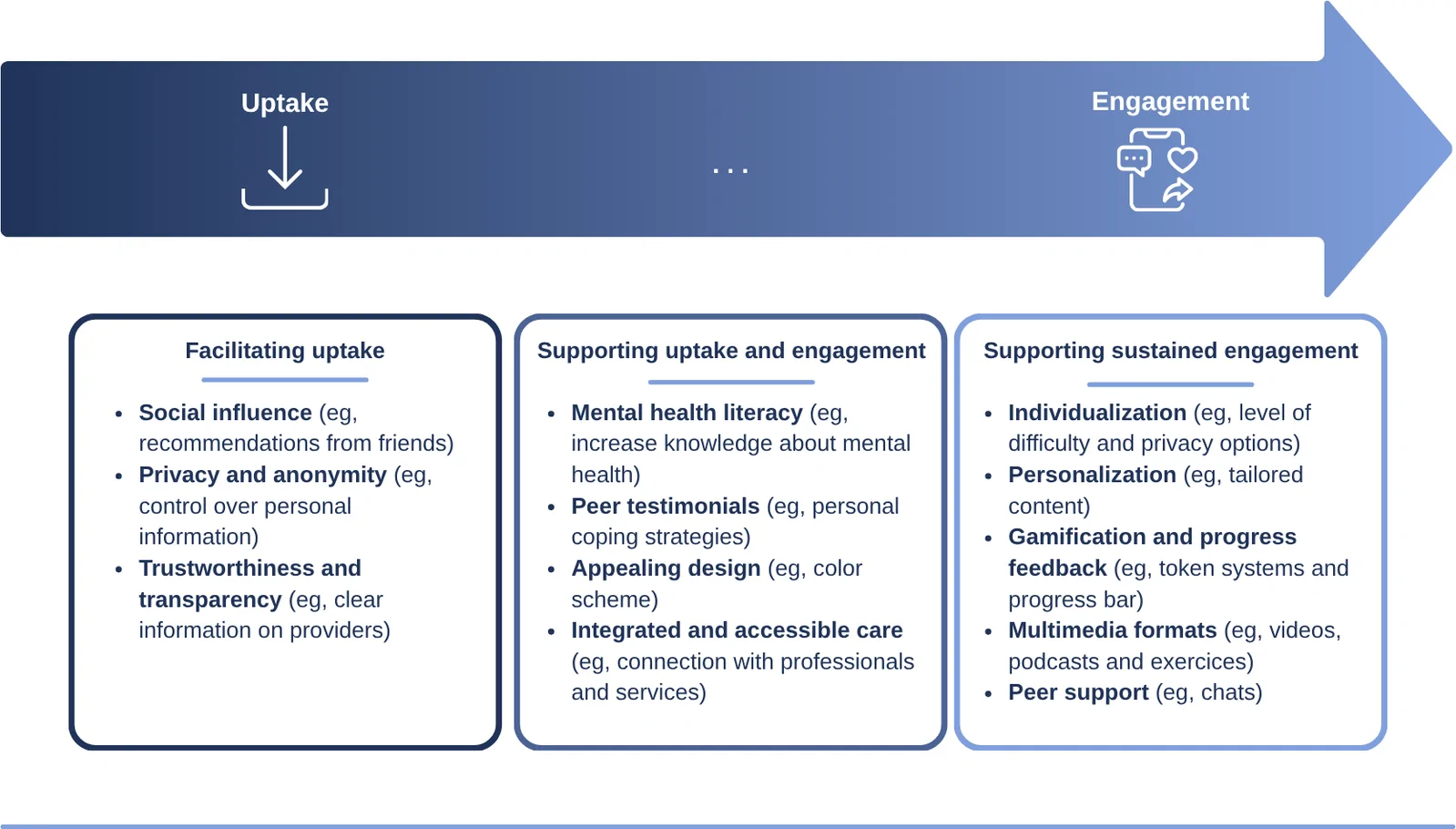
Conceptual overview of implementation suggestions according to their primary contribution to uptake, engagement, or both in digital mental health interventions. Detailed implementation recommendations are provided in Multimedia Appendix 5.

### System Level

#### Ideas and Wishes for Adaptive, Integrated, and Low-Threshold Mental Health Services

Participants’ expectations extended beyond the design of individual DMHIs to the way DMHIs should be embedded within the wider mental health services to better meet their needs. They envisioned mental health care systems that are adaptive in intensity, provide integrated services, and offer low-threshold access. This topic emerged inductively and was not anticipated by the theoretical framework.

Participants expected DMHIs to raise awareness of mental health problems and to provide different levels of support depending on users’ perceived symptom severity. While mild problems were generally considered appropriate for self-management or support from friends and family, professional support was viewed as more appropriate for more severe symptoms. Specifically, participants described an approach of graduated care with (1) digital psychoeducation to improve mental health literacy, (2) self-guided preventive DMHI use, (3) proactive outreach by the DMHI if symptoms deteriorate, (4) flexible digital or face-to-face counseling access based on users’ own judgments, and (5) an emergency button for immediate professional support. Notably, participants’ descriptions closely resembled a stepped-care model, despite not having been introduced to this concept.

*[It would be helpful to] know: “okay, from these symptoms onward or whatever…” might be the point where one should seek professional help instead of relying solely on the program. But in general, the aim of the program is not seeking help from a person but to be able to solve [your problems] online on your own. So, a note [about available services] would be important but not that they are a part of [the intervention]*.

[TN42, 18 years, female]

Participants strongly preferred all services to be embedded within the DMHI rather than provided as external links, including appointment booking with local professionals and peer support networks. They also appreciated having different communication options, such as chat, email, video consultations, forums, or digital self-help groups.

*[...] because if you realize - based on the information or maybe some tips provided there - something like, “If you’re feeling this way, it might be good to see a psychologist or psychiatrist,” I would find it very helpful if that option were offered directly within the program. That way, you wouldn’t have to search for someone on your own*.

[TN7, 21 years, female]

Finally, low-threshold access to DMHIs and just-in-time, continuous support for adolescents in contexts of acute need was considered essential, with participants repeatedly noting that mandatory registration or personal data requirements at uptake should be minimized. This was expected to simplify navigation through the mental health care system and reduce the effort associated with accessing appropriate support.

### Cross-Level Themes

#### Trust

Participants emphasized anonymous use, information about the credibility of the provider, and testimonials highlighting the usefulness of the intervention as important facilitators of uptake. Although these aspects initially emerged as intervention-level design requirements, further analysis showed that they reflected the broader concept of trust spanning person-, intervention-, and system-level factors. At the person level, trust influenced adolescents’ willingness to disclose personal information and discuss their mental health challenges. At the intervention level, trust was fostered through transparent information about the provider, options for anonymous use, and testimonials from other users. At the system level, participants expected the intervention to be embedded within and endorsed by trusted institutions, such as universities or health care providers, which enhanced its perceived credibility.

*I feel like it should clearly state where it comes from, who is behind it and what their motivation is for creating it. To me, seeing that it’s an intervention from a university and that there are studies behind it, that would definitely create a sense of trustworthiness. And I feel like it needs a clear statement like “About us” and a way for me to associate [the intervention] with something trustworthy and legitimate, or otherwise there should be some way to demonstrate why [the intervention] is trustworthy*.

[TN10, 17 years, male]

Trust further encompassed expectations regarding data security and the handling of personal information. Participants expected transparent information about data protection to be presented prominently at the beginning of the DMHI, including clear explanations of what personal data would be collected, how they would be used, and who would have access to them. Beyond the initial registration process, participants preferred additional consent to be obtained before storing further sensitive information, allowing them to retain control over the disclosure of personal data. Together, these findings suggest that trust was not perceived as a discrete intervention feature but as a prerequisite for expected engagement shaped jointly by privacy concerns, intervention design, and institutional credibility.

#### Support, Reassurance, and Connection

A second cross-level theme concerned adolescents’ need for support, reassurance, and connection. Although support expectations were initially captured as intervention-level features, analysis revealed that the experience of feeling supported could not be adequately described at a single level; it was shaped simultaneously by personal needs for validation and connection, by specific intervention characteristics, and by expectations regarding the wider mental health care system.

At the person level, participants described a fundamental need to feel that they were not alone with their difficulties. Knowing that others shared similar experiences was expected to reduce feelings of isolation and normalize mental health challenges, thereby increasing willingness to engage with the intervention.

At the intervention level, 3 complementary expectations emerged. First, participants expected help to be accessible when needed—either through immediate support in moments of acute distress or simply through the reassurance that professional help was available if required. Crucially, participants consistently preferred access to a real professional rather than AI-based support, as human availability was perceived as more reassuring and trustworthy. This preference for human over AI support emerged inductively.

*And if I knew that in an emergency, I could get help or whatever [on] there every evening it would provide me with a huge sense of reassurance in that moment*.

[TN5, 21 years, female]

Second, participants valued opportunities to learn that others experienced similar difficulties through peer exchange. However, views differed regarding the desired level of interaction; passive engagement, such as reading others’ experiences, was broadly accepted, whereas active self-disclosure was viewed more critically due to concerns about negative social comparisons, symptom reinforcement, or becoming overly focused on supporting others at the expense of one’s own well-being.

Third, participants emphasized the importance of feeling seen, understood, and taken seriously, which is also an inductively derived expectation not anticipated in the predefined feature list. They expected professionals to demonstrate genuine interest in their individual situation and the intervention itself to communicate empathy through its tone, content, and interactions.

*[I would need to know] if the person [on the other side] is genuinely interested in me. [And I would like] to feel like I am being taken seriously for real and to feel like the questions and content [in the DMHI] are applicable to my situation*.

[TN15, 19 y, female]

At the system level, the continuous availability and integration of services described in the preceding section did not function merely as practical conveniences but as signals of institutional commitment. Participants’ sense of being genuinely supported was contingent not only on what the DMHI itself offered but on whether it was embedded within a care system that would remain available if their needs changed. Together, these findings suggest that adolescents did not perceive support as a discrete intervention feature but as an experience emerging from the interaction between individual needs, intervention design, and the wider institutional context in which the DMHI is integrated.

#### Discrepancies Between Participants’ Expectations and Implementation Possibilities

The third cross-level theme concerned tensions between adolescents’ desired characteristics of preventive DMHIs and the practical requirements and limitations of implementation. These tensions emerged through the following subtopics: (1) wish to share as little personal information as possible while also valuing personalized support, (2) interest in reading about others’ experiences while simultaneously recognizing the stigma around mental health and avoiding self-disclosure, and (3) the wish for 24/7, immediate, local support despite a preference for minimal personal engagement. While these tensions are not necessarily incompatible from a provider’s perspective, integrating them into a single service can be challenging or even impossible. Providing personalized support becomes more challenging when professionals are expected to be familiar with users’ personal information or when users facing similar challenges should be connected in a chat room - without requiring a registration or login process to store user data. Some participants realized this discrepancy and even stated a registration process as a feature that made a DMHI more trustworthy.

*[I don’t necessarily have ideas to implement data security.] That’s why I didn’t really like the idea of creating profiles, because they reveal a lot of information. [While] data protection is important, in case of an emergency, e-coaches should still be able to access basic data like an address or name if needed*.

[TN38, 18 y, female]

As mentioned before, peer exchange was highly appreciated. However, in line with the wish for anonymity and the awareness of stigma surrounding mental health issues, participants often hesitated to express a willingness to actively engage in these interactions and to openly share their own experiences. Instead, passive participation, such as reading about others’ experiences, was more commonly preferred.

*I think it’s important that [exchange with other adolescents] exists. But for me, it’s always a bit uncomfortable to actually participate in the exchange. However, I do like reading when others share their experiences*.

[TN18, 21 years, female]

While many participants expressed the wish for constant availability of help resulting in immediate personal responses and improvement of mental health symptoms, they also frequently emphasized the importance of locally embedded services. When asked about how long and how often the participants would engage with a DMHI, answers ranged from a couple of minutes to 2 hours per login. Moreover, the willingness to use a DMHI over a longer period ranged from a couple of days to four years if it is perceived as being helpful. The identified discrepancy therefore reflects the contrast between availability and locality of the provider and between the experienced mental health benefits and willingness to engage with the DMHI for a very limited amount of time.

## Discussion

### Principal Findings

This study is among the first to qualitatively investigate adolescents’ perspectives on factors influencing the anticipated uptake and expected engagement with transdiagnostic preventive DMHIs. Using a hybrid deductive-inductive approach, we identified factors operating within and across person, intervention, and system levels. Beyond level-specific considerations, trust, support, and discrepancies between adolescents’ expectations and implementation possibilities emerged as important cross-level themes shaping how preventive DMHIs are perceived and how they may be used.

### Relevance of Awareness and Preuptake Communication

The findings of this study suggest that adolescents’ decision to engage with a preventive DMHI begins well before they encounter the intervention itself. Person-level factors were particularly relevant during the awareness phase of uptake, highlighting the importance of mental health literacy and social contexts that facilitate recognition of and access to available support options. These findings align with previous research on help-seeking and DMHI engagement and correspond to the capability and opportunity domains of the COM-B model [2,3,61,62].

A noteworthy finding was that adolescents’ informational needs extended beyond general mental health literacy. Before considering uptake, they required concrete information about the intervention’s purpose, credibility, expected commitment, and access procedures. This suggests that uptake barriers may arise before first contact with the intervention and highlights the importance of targeted preuptake communication. Furthermore, the prominent role of peers and family members as sources of awareness suggests that adolescents’ social networks may represent an underused pathway for disseminating preventive DMHIs. However, because this reflects hypothetical rather than observed dissemination, relying on peer- and family-based promotion could also disadvantage adolescents with weaker social support or in more stigmatizing environments.

Perceived usefulness and anticipated stigma further shaped adolescents’ willingness to engage with preventive DMHIs. These findings are consistent with previous research identifying perceived usefulness as a key determinant of DMHI uptake [63] and align with the health belief model, which proposes that factors such as knowledge and previous experiences influence health-related decision-making by shaping beliefs about benefits, barriers, and the personal relevance of preventive interventions [57,64]. Thus, adolescents’ willingness to use DMHIs may depend not only on awareness of available interventions, but also on whether they perceive them as meaningful, applicable, and worthwhile in relation to their own needs and concerns.

### Intervention and System Requirements for Uptake and Sustained Engagement

Once adolescents considered using a preventive DMHI, their expectations shifted from whether to use the intervention to whether it met their expectations regarding quality, usability, and accessibility. A key finding was that participants evaluated preventive DMHIs against the standards of commercial digital applications rather than traditional health care services. Consequently, appealing design, intuitive navigation, and affordability appeared to function as prerequisites for uptake rather than features that actively promote engagement. While these findings are consistent with previous research on facilitators of DMHI engagement [3,12,16], they additionally highlight the importance of distinguishing between factors influencing initial uptake and those supporting sustained engagement.

Regarding content of the DMHI, participants further emphasized the importance of reducing cognitive burden during engagement. Consistent with previous research on usability and suitability [16], adolescents valued concise, accessible content and inclusive representation rather than demographic segmentation. Personalization and individualization were also considered important for enhancing engagement as in previous research [16,65], but only when combined with user control over data disclosure. This highlights that personalization cannot be considered independently from privacy and autonomy [66], a tension further reflected in the cross-level discrepancies identified in this study.

Beyond individual intervention characteristics, participants envisioned preventive DMHIs as integrated components of adaptive and low-threshold mental health care systems. A particularly noteworthy finding was that adolescents spontaneously described a stepped-care approach, including different levels of digital and professional support depending on individual needs, despite not having been introduced to this concept. This convergence with established stepped-care models [58-60] suggests that adolescents may intuitively recognize the value of flexible care pathways and provides further support for embedding preventive DMHIs within broader health care structures rather than positioning them as standalone tools. Participants also highlighted the potential value of integrated peer and human support options, suggesting that social connection may represent an important component of continuity within digital mental health care [60]. However, as participants favored passive over active peer exchange, such features would need moderation to safeguard against harmful peer advice and negative social comparison.

Notably, although the study focused on preventive, subthreshold DMHIs, participants frequently described expectations more characteristic of clinical or crisis-oriented services, including immediate professional availability and emergency support. This may reflect difficulties in conceptualizing prevention as an early support strategy and in distinguishing preventive support from early intervention, treatment, and crisis care, extending existing concerns about mental health literacy [67,68] to the domain of digital prevention. Clear communication about the purpose and boundaries of preventive DMHIs may therefore be essential to support appropriate uptake and engagement. In particular, preventive DMHIs may benefit from psychoeducational information that clarifies what the intervention can provide, as well as clear pathways for accessing professional or crisis-oriented support when needed.

### Trust, Support, and the Interaction Between Levels

Beyond factors operating within individual levels, trust and support highlighted that the willingness to try and engage with preventive DMHIs emerges through interactions between person-, intervention-, and system-level factors. Although initially identified through specific intervention expectations, both concepts extended beyond the boundaries of a single analytical level. Trust was not merely a feature of the intervention but a prerequisite for engagement shaped by adolescents’ willingness to disclose information, perceptions of transparency and privacy, and confidence in the institutions delivering the intervention. Similarly, support was not perceived as an isolated intervention component but as an experience emerging from the interaction between individual needs, intervention characteristics, and available care structures.

Interestingly, while both human- and AI-supported DMHIs have been shown to enhance engagement [12,19,69], participants in this study expressed a clear preference for human support. This preference suggests that adolescents may not perceive support solely as an intervention feature intended to increase engagement, but also as a relational component providing reassurance, validation, and a sense of being taken seriously. For preventive DMHIs, this highlights the importance of clearly defining and communicating the role of human involvement: while access to professional support may strengthen perceived safety, unlimited availability of individual professional contact may conflict with the preventive and self-guided nature of these interventions. Clear boundaries around the availability and role of human support, together with defined escalation pathways for situations requiring more intensive care, may therefore be important to avoid unrealistic expectations of continuous or 24/7 professional support. These findings suggest that trust and support cannot be achieved through intervention design alone but also depend on transparent communication and credible integration within trusted health care structures. Future development should also consider the operational feasibility of human support, including staffing requirements, legal liability, and sustainable funding mechanisms, as key implementation questions for integrating human support into preventive DMHIs.

### Discrepancies Between Support Expectations and Preventive Aims

This study identified three recurring tensions between adolescents’ expectations of preventive DMHIs and the practical realities of implementation: the desire for personalized support while minimizing personal data disclosure, the wish to benefit from peers’ experiences without active self-disclosure, and the expectation of immediate human support despite limited intended engagement with the intervention. Rather than representing contradictions, these findings reflect competing priorities that require careful balancing during intervention development.

These findings have important implications for the development of future DMHIs and their implementation. Clear communication at the point of uptake regarding the intervention’s purpose, expected level of engagement, and handling of personal data may help align users’ expectations with the preventive aims of the intervention. Similarly, the balance between anonymity and personalization should be guided by the primary objective of the intervention, as greater anonymity may reduce barriers to uptake whereas more personalized support may require increased disclosure [70]. Participants’ own implementation suggestions reflected this tension directly, ranging from anonymity-related requests such as optional data provision and the use of nicknames to personalization-related requests such as customizable privacy settings within individualized profiles (Multimedia Appendix 5). The expectation of continuous human support warrants particular attention because it conflicts with the preventive and self-guided nature of DMHIs. Preventive interventions should therefore clearly communicate their scope and limitations, provide transparent pathways to higher-intensity care when needed, and position human support as an escalation pathway rather than a substitute for professional mental health services. Specifically, these findings informed the subsequent design of the EMPATIA intervention. Adolescents’ need for concrete information before uptake informed the inclusion of clear and prominently placed information about the intervention’s content, duration, and provider/source. Their preference for accessible human support was addressed by the inclusion of human e-guidance. Furthermore, participants’ preferences for individualized support informed the use of self-assessment exercises at the beginning of modules to support the selection of relevant modules, strategies, and examples.

### Limitations

Several limitations should be considered when interpreting these findings. First, the sample was predominantly female (73%) and highly educated, reflecting a self-selecting sample of adolescents willing to participate in mental health research. Consequently, the findings may not generalize to male adolescents, those with lower educational attainment, migrant or minority-language backgrounds, or adolescents with little prior experience of mental health support, with no or little interest in mental health topics, or with low trust in health or academic institutions. Furthermore, race, ethnicity, migration background, and socioeconomic status were not assessed, and eligibility was restricted to adolescents residing in Switzerland who understood and spoke German. This may have excluded the perspectives of multilingual, migrant, or otherwise minoritized youth. In addition, although age and gender were recorded, participants may have differed substantially in developmental stage, autonomy, privacy expectations, help-seeking, and parental involvement, which may have shaped their interview responses. However, the sample composition did not allow meaningful exploration of age-related or other subgroup differences, including the potential influence of intersecting social identities. Moreover, prior mental health symptoms and help-seeking experiences were not related to the qualitative findings, limiting interpretation of whether participants’ perspectives varied according to their prior mental health experience. Future research should therefore include more diverse samples and examine developmental differences in participants’ perspectives.

Second, the limited prior experience of the master’s student interviewers in qualitative research methods represents a potential limitation. Although this was mitigated through structured preparation, pilot interviews, reflexive discussions, and ongoing expert supervision, future studies may benefit from involving interviewers with more extensive qualitative research experience.

Third, shared assumptions about digital engagement between the master’s student interviewers and participants may have limited the exploration of certain topics during follow-up questioning. Furthermore, the all-female research team may have influenced data interpretation. These potential influences were addressed through reflexive diary-keeping, regular discussions within the research team, and repeated review of coding and interpretations.

Finally, the semistructured, theory-derived interview guide and initial coding framework may have influenced participants’ responses by directing attention toward elements of the research team’s proposed intervention and established theoretical frameworks [36]. Although the analysis allowed inductive themes to emerge, the influence of these predefined starting points cannot be excluded. Moreover, this study focused on the discovery phase of co-design rather than involving adolescents throughout the full design process. Future co-design efforts should therefore involve adolescents throughout all stages of intervention development and evaluation [27,30,31,39].

### Conclusion

This study identified person-, intervention-, system-, and cross-level factors shaping adolescents’ anticipated uptake and expected engagement with preventive transdiagnostic DMHIs during the predesign phase of intervention development. At the person level, targeted preuptake communication addressing adolescents’ informational needs and leveraging social networks may facilitate uptake. At the intervention level, high-quality design and a balanced approach to personalization that preserves user control over data disclosure may support both uptake and sustained engagement. At the system level, adolescents’ descriptions of adaptive, low-threshold, and integrated mental health services highlight the importance of embedding preventive DMHIs within broader care pathways rather than positioning them as standalone tools. Beyond these level-specific findings, participants’ difficulties conceptualizing prevention suggest that implementation should be accompanied by psychoeducational efforts that clarify the purpose of preventive DMHIs and distinguish them from crisis and treatment services. Moreover, the tension between adolescents’ desire for immediate human support and the preventive, self-guided nature of these interventions highlights the need for clear clinical models defining the role and limits of digital preventive support. These findings should be understood as generating implementation hypotheses to be tested in subsequent phases of intervention development. Furthermore, they emphasize that successful implementation of preventive DMHIs requires coordinated attention to adolescents’ needs, intervention design, and the wider health care context.

## Supplementary material

10.2196/76494 Multimedia Appendix 1 Interview guidelines (original version).

10.2196/76494 Multimedia Appendix 2 Importance ratings of predefined intervention features.

10.2196/76494 Multimedia Appendix 3 Extended author reflexivity statement.

10.2196/76494 Multimedia Appendix 4 Codebook with main topics, subtopics, code descriptions, and representative quotes.

10.2196/76494 Multimedia Appendix 5 Detailed implementation suggestions.

## Abbreviations

AHSQ: Actual Help Seeking Questionnaire
COM-B: capability-opportunity-motivation behavior
COREQ: Consolidated criteria for Reporting Qualitative research
DMHI: digital mental health intervention
GHSQ: General Help Seeking Questionnaire
K10: Kessler Psychological Distress Scale

## References

[1] Solmi M, Radua J, Olivola M (2022). Age at onset of mental disorders worldwide: large-scale meta-analysis of 192 epidemiological studies. Mol Psychiatry.

[2] Aguirre Velasco A, Cruz ISS, Billings J, Jimenez M, Rowe S (2020). What are the barriers, facilitators and interventions targeting help-seeking behaviours for common mental health problems in adolescents? A systematic review. BMC Psychiatry.

[3] Huff NR, Dunderdale L, Kellogg AJ, Isbell LM (2024). Factors related to help-seeking and service utilization for professional mental healthcare among young people: an umbrella review. Clin Psychol Rev.

[4] Radez J, Reardon T, Creswell C, Lawrence PJ, Evdoka-Burton G, Waite P (2021). Why do children and adolescents (not) seek and access professional help for their mental health problems? A systematic review of quantitative and qualitative studies. Eur Child Adolesc Psychiatry.

[5] Westberg KH, Nyholm M, Nygren JM, Svedberg P (2022). Mental health problems among young people-a scoping review of help-seeking. Int J Environ Res Public Health.

[6] Cavelti M, Ruppen NA, Sele S (2025). An examination of sociodemographic and clinical factors influencing help-seeking attitudes and behaviors among adolescents with mental health problems. Eur Child Adolesc Psychiatry.

[7] Lustig S, Kaess M, Schnyder N (2023). The impact of school-based screening on service use in adolescents at risk for mental health problems and risk-behaviour. Eur Child Adolesc Psychiatry.

[8] Kazdin AE (2023). Addressing the treatment gap: expanding the scalability and reach of treatment. J Consult Clin Psychol.

[9] Werlen L, Gjukaj D, Mohler-Kuo M, Puhan MA (2020). Interventions to improve children’s access to mental health care: a systematic review and meta-analysis. Epidemiol Psychiatr Sci.

[10] Sweeney GM, Donovan CL, March S, Forbes Y (2019). Logging into therapy: adolescent perceptions of online therapies for mental health problems. Internet Interv.

[11] Pretorius C, Chambers D, Coyle D (2019). Young people’s online help-seeking and mental health difficulties: systematic narrative review. J Med Internet Res.

[12] Opie JE, Vuong A, Welsh ET, Esler TB, Khan UR, Khalil H (2024). Outcomes of best-practice guided digital mental health interventions for youth and young adults with emerging symptoms: part II. A systematic review of user experience outcomes. Clin Child Fam Psychol Rev.

[13] Wright M, Reitegger F, Cela H, Papst A, Gasteiger-Klicpera B (2023). Interventions with digital tools for mental health promotion among 11-18 year olds: a systematic review and meta-analysis. J Youth Adolesc.

[14] Zhou X, Edirippulige S, Bai X, Bambling M (2021). Are online mental health interventions for youth effective? A systematic review. J Telemed Telecare.

[15] d’Halluin A, Costa M, Morgiève M, Sebbane D (2023). Attitudes of children, adolescents, and their parents toward digital health interventions: scoping review. J Med Internet Res.

[16] Liverpool S, Mota CP, Sales CMD (2020). Engaging children and young people in digital mental health interventions: systematic review of modes of delivery, facilitators, and barriers. J Med Internet Res.

[17] Achilles MR, Anderson M, Li SH, Subotic-Kerry M, Parker B, O’Dea B (2020). Adherence to e-mental health among youth: considerations for intervention development and research design. Digit Health.

[18] Pretorius C, McCashin D, Kavanagh N, Coyle D (2020). CHI ’20: Proceedings of the 2020 CHI Conference on Human Factors in Computing Systems.

[19] Boucher EM, Raiker JS (2024). Engagement and retention in digital mental health interventions: a narrative review. BMC Digit Health.

[20] Jardine J, Nadal C, Robinson S, Enrique A, Hanratty M, Doherty G (2024). Between rhetoric and reality: real-world barriers to uptake and early engagement in digital mental health interventions. ACM Trans Comput-Hum Interact.

[21] Taylor CB, Ruzek JI, Fitzsimmons-Craft EE (2020). Using digital technology to reduce the prevalence of mental health disorders in populations: time for a new approach. J Med Internet Res.

[22] Michie S, Atkins L, West R (2014). The Behaviour Change Wheel: A Guide to Designing Interventions.

[23] Lipschitz JM, Pike CK, Hogan TP, Murphy SA, Burdick KE (2023). The engagement problem: a review of engagement with digital mental health interventions and recommendations for a path forward. Curr Treat Options Psychiatry.

[24] Mora Ringle VA, Sung JY, Roulston CA, Schleider JL (2024). Mixed-methods examination of adolescent-reported barriers to accessing mental health services. J Adolesc Health.

[25] Roulston CA, Ahuvia I, Chen S, Fassler J, Fox K, Schleider JL (2025). “My family won’t let me.” Adolescent-reported barriers to accessing mental health care. J Res Adolesc.

[26] Moltrecht B, Patalay P, Bear HA, Deighton J, Edbrooke-Childs J (2022). A transdiagnostic, Emotion Regulation App (Eda) for children: design, development, and lessons learned. JMIR Form Res.

[27] Thabrew H, Fleming T, Hetrick S, Merry S (2018). Co-design of eHealth interventions with children and young people. Front Psychiatry.

[28] Bear HA, Ayala Nunes L, Ramos G (2024). The acceptability, engagement, and feasibility of mental health apps for marginalized and underserved young people: systematic review and qualitative study. J Med Internet Res.

[29] Biernesser C, Win E, Escobar-Viera C, Farzan R, Rose M, Goldstein T (2023). Development and codesign of flourish: a digital suicide prevention intervention for LGBTQ+ youth who have experienced online victimization. Internet Interv.

[30] Malloy J, Partridge SR, Kemper JA, Braakhuis A, Roy R (2023). Co-design of digital health interventions with young people: a scoping review. Digit Health.

[31] Bevan Jones R, Stallard P, Agha SS (2020). Practitioner review: co-design of digital mental health technologies with children and young people. J Child Psychol Psychiatry.

[32] Ludlow K, Russell JK, Ryan B (2023). Co-designing a digital mental health platform, “momentum”, with young people aged 7-17: a qualitative study. Digit Health.

[33] Baka E, Tan YR, Wong BLH, Xing Z, Yap P (2025). A scoping review of digital interventions for the promotion of mental health and prevention of mental health conditions for young people. Oxf Open Digit Health.

[34] Hartmann JA, McGorry PD, Destree L (2020). Pluripotential risk and clinical staging: theoretical considerations and preliminary data from a transdiagnostic risk identification approach. Front Psychiatry.

[35] McGorry PD, Hartmann JA, Spooner R, Nelson B (2018). Beyond the "at risk mental state" concept: transitioning to transdiagnostic psychiatry. World Psychiatry.

[36] Hirsig A, Häfeli XA, Schmidt SJ (2024). Efficacy of a transdiagnostic Internet prevention approach in adolescents (EMPATIA study): study protocol of a randomized controlled trial. Trials.

[37] Egan SJ, Neal J, Ure S (2023). The development of co-designed parent-supported cognitive behaviour therapy for perfectionism in adolescents with eating disorders: initial feasibility and acceptability. J Eat Disord.

[38] Moghimi E, Belfry K, Farr S (2025). Using a co-design approach to develop a Preventative Online Mental Health Program for Youth (POMHPY): a quality improvement project. BMC Health Serv Res.

[39] Sanders EBN, Stappers PJ (2014). Probes, toolkits and prototypes: three approaches to making in codesigning. CoDesign.

[40] Baines R, Bradwell H, Edwards K (2022). Meaningful patient and public involvement in digital health innovation, implementation and evaluation: a systematic review. Health Expect.

[41] Orlowski SK, Lawn S, Venning A (2015). Participatory research as one piece of the puzzle: a systematic review of consumer involvement in design of technology-based youth mental health and well-being interventions. JMIR Hum Factors.

[42] Tong A, Sainsbury P, Craig J (2007). Consolidated Criteria for Reporting Qualitative Research (COREQ): a 32-item checklist for interviews and focus groups. Int J Qual Health Care.

[43] Baird S, Choonara S, Azzopardi PS (2025). A call to action: the Second Lancet Commission on adolescent health and wellbeing. Lancet.

[44] Sawyer SM, Azzopardi PS, Wickremarathne D, Patton GC (2018). The age of adolescence. Lancet Child Adolesc Health.

[45] Alessi EJ, Kahn S (2023). Toward a trauma-informed qualitative research approach: guidelines for ensuring the safety and promoting the resilience of research participants. Qual Res Psychol.

[46] Draucker CB, Martsolf DS, Poole C (2009). Developing distress protocols for research on sensitive topics. Arch Psychiatr Nurs.

[47] Whitney C, Evered JA (2022). The qualitative research distress protocol: a participant-centered tool for navigating distress during data collection. Int J Qual Methods.

[48] Malterud K (2001). The art and science of clinical knowledge: evidence beyond measures and numbers. Lancet.

[49] Castillo-Montoya M (2016). Preparing for interview research: the interview protocol refinement framework. Qual Rep.

[50] Wilson CJ, Deane FP, Ciarrochi J, Rickwood D (2007). Measuring help-seeking intentions: properties of the general help seeking questionnaire. Can J Couns Psychother.

[51] Rickwood DJ, Braithwaite VA (1994). Social-psychological factors affecting help-seeking for emotional problems. Soc Sci Med.

[52] Kessler RC, Andrews G, Colpe LJ (2002). Short screening scales to monitor population prevalences and trends in non-specific psychological distress. Psychol Med.

[53] Kuckartz U (2018). Qualitative Content Analysis: Methods, Practice, Computer Support [Book in German].

[54] Kuckartz U, Rädiker S (2023). Qualitative Content Analysis: Methods, Practice and Software.

[55] (2024). The #1 qualitative data analysis software with the best AI integration. MAXQDA.

[56] Levesque JF, Harris MF, Russell G (2013). Patient-centred access to health care: conceptualising access at the interface of health systems and populations. Int J Equity Health.

[57] Janz NK, Becker MH (1984). The Health Belief Model: a decade later. Health Educ Q.

[58] Alvarez-Jimenez M, Nicholas J, Valentine L (2025). A national evaluation of a multi-modal, blended, digital intervention integrated within Australian youth mental health services. Acta Psychiatr Scand.

[59] Mughal S, Young B, Churchill A (2025). Proposed standards for implementing stepped care models in child and youth mental health service systems: results of a pan-Canadian Delphi study. Authorea.

[60] Ridout B, Forsyth R, Amon KL, Navarro P, Campbell AJ (2024). The urgent need for an evidence-based digital mental health practice model of care for youth. JMIR Ment Health.

[61] Baldofski S, Scheider J, Kohls E (2024). Intentions and barriers to help-seeking in children and adolescents differing in depression severity: cross-sectional results from a school-based mental health project. Research Square.

[62] Stiles-Shields C, Ramos G, Ortega A, Psihogios AM (2023). Increasing digital mental health reach and uptake via youth partnerships. Npj Mental Health Res.

[63] Zhu S, Wang Y, Hu Y (2025). Facilitators and barriers to digital mental health interventions for depression, anxiety, and stress in adolescents and young adults: scoping review. J Med Internet Res.

[64] Wethington E, Glanz K, Schwartz MD, Glanz K, Rimer BK, Viswanath KV (2015). Health Behavior: Theory, Research, and Practice.

[65] Valentine L, Arnold C, Nicholas J (2024). A personalized, transdiagnostic smartphone app (Mello) targeting repetitive negative thinking for depression and anxiety: qualitative analysis of young people’s experience. J Med Internet Res.

[66] Zhang R, Ringland KE, Paan M, Mohr DC, Reddy M (2021). CHI ’21: Proceedings of the 2021 CHI Conference on Human Factors in Computing Systems.

[67] Bergin AD, Vallejos EP, Davies EB (2020). Preventive digital mental health interventions for children and young people: a review of the design and reporting of research. NPJ Digit Med.

[68] Mills J, Cumbers L, Williams S, Titley-Wall H (2023). The effectiveness of mental health literacy interventions in young people: a meta-analysis. Ment Health Rev J.

[69] Boucher EM, Ward H, Miles CJ, Henry RD, Stoeckl SE (2024). Effects of a digital mental health intervention on perceived stress and rumination in adolescents aged 13 to 17 years: randomized controlled trial. J Med Internet Res.

[70] Borghouts J, Eikey E, Mark G (2021). Barriers to and facilitators of user engagement with digital mental health interventions: systematic review. J Med Internet Res.

