# Supplementary material for: Adolescents’ Perspectives on Uptake, Engagement, and Implementation to Inform the Development of a Preventive Transdiagnostic Digital Mental Health Intervention: Qualitative Interview Study
Source: J Med Internet Res. 2026 Nov 2;28:e76494. doi: 10.2196/76494 (PMC13727026; doi:10.2196/76494)
Supplement: Supplementary file 1 [file jmir-v28-e76494-s001.pdf]

Leitfaden semistrukturierte Interviews der BePart-Studie

Willkommen und Einleitung

Herzlich Willkommen zu unserem Interview im Rahmen der BePart-Studie. Wir freuen uns sehr über Deine Teilnahme. Mit Deiner Hilfe können wir besser verstehen, was die Bedürfnisse junger Leute sind und was sie sich für ein Online-Programm zur Förderung der psychischen Gesundheit von Jugendlichen wünschen würden. Deine Meinung ist uns dafür sehr wichtig!
Das Interview dauert etwa 30-45 Minuten. Bei den Fragen gibt es keine richtigen oder falschen Antworten; antworte einfach spontan. Falls Du eine Frage nicht beantworten möchtest, kannst Du mir das gerne sagen.
Deine Antworten werden ohne Deinen Namen auf Audio gespeichert, so dass wir sie danach analysieren können, ohne dass wir wissen, dass dies Deine Antwort war.
Hast Du irgendwelche Fragen?
Dann starte ich jetzt das Audio und wir beginnen mit ein paar Fragen zu Deiner Person.

Soziodemographische Angaben

- ***Alter:*** ____________

- ***Geschlecht***
    - *weiblich*
    - *männlich*
    - *anderes*

- ***Postleitzahl Deines Wohnortes****:* ____________

- ***Mit wem lebst Du im selben Haushalt?*** ____________

- ***Hast Du ein eigenes Schlafzimmer?*** ____________

- ***Wie viel Zeit verbringst Du pro Tag*** **(während der Woche und am Wochenende)** ***aktiv im Internet?*** ____________

- ***Welche Schule besuchst Du gerade bzw. hast Du zuletzt besucht?***
    - *keinen Schulabschluss oder Schule abgebrochen*
    - *Sonderschule*
    - *Primarschule*
    - *Realschule*
    - *Sekundarschule*
    - *10. Schuljahr*
    - *Gymnasium*
    - *Berufsschule/Gewerbeschule*
    - *Berufsmaturität*
    - *Mittelschule*
    - *andere Schule:* __________

- ***In welche Klasse gehst Du aktuell? __________***

- ***Hast Du die Schule bereits abgeschlossen?***
    - *Ja und ich bin gerade in einer Lehre/Ausbildung*
    - *Ja und ich bin gerade in einem Zwischenjahr/Brückenjahr*
    - *Ja und ich bin gerade in einem Praktikum*
    - *Ja und ich habe meine Lehre/Ausbildung bereits abgeschlossen*
    - *Ja und ich studiere gerade an einer Universität oder Fachhochschule*
    - *Ja und ich bin gerade arbeitssuchend*
    - *Nein, ich bin noch in der Schule*
    - *Anderes: __________________*

- *Was ist der höchste Schulabschluss Deiner Mutter?*
    - *Keinen Abschluss*
    - *Sonderschulabschluss*
    - *Primarschulabschluss (6 Jahre)*
    - *Realschulabschluss (9 Jahre)*
    - *Sekundarschulabschluss (9 Jahre)*
    - *10. Schuljahr*
    - *Gymnasium (Berufs- Maturität)*
    - *Mittelschule*
    - *Anderer Schulabschluss*
    - *nicht bekannt*

- *Was ist der höchste Berufsabschluss Deiner Mutter?*
    - *Keinen Berufsabschluss*
    - *Mutter? Anlehre (max. 2 Jahre)*
    - *Lehre oder gleichwertiger Abschluss (3 bis 4 Jahre)*
    - *Meister / Berufsprüfung*
    - *(Fach-)Hochschule / Universität*
    - *Doktorat*
    - *Habilitation*
    - *anderer Berufsabschluss*
    - *nicht bekannt*

- *Was ist der höchste Schulabschluss Deines Vaters?*
    - *Keinen Abschluss*
    - *Sonderschulabschluss*
    - *Primarschulabschluss (6 Jahre)*
    - *Realschulabschluss (9 Jahre)*
    - *Sekundarschulabschluss (9 Jahre)*
    - *10. Schuljahr*
    - *Gymnasium (Berufs- Maturität)*
    - *Mittelschule*
    - *Anderer Schulabschluss*
    - *nicht bekannt*

- *Was ist der höchste Berufsabschluss Deines Vaters?*
    - *Keinen Berufsabschluss*
    - *Mutter? Anlehre (max. 2 Jahre)*
    - *Lehre oder gleichwertiger Abschluss (3 bis 4 Jahre)*
    - *Meister / Berufsprüfung*
    - *(Fach-)Hochschule / Universität*
    - *Doktorat*
    - *Habilitation*
    - *anderer Berufsabschluss*
    - *nicht bekannt*

Als Nächstes kommen wir zu Fragen, die direkt unsere Studie betreffen.

Erfahrung mit Online-Programmen

1. *Gibt es Websiten, Online-Programme oder Apps, die Du regelmässig verwendest?*
    a. *Wie heissen sie?*
    b. *Was gefällt Dir daran und was gefällt Dir nicht?*

2.
    a. *Wo würdest Du Dich online über psychische Probleme informieren und ggf. Hilfe suchen?*
    b. *Welche Suchbegriffe würdest Du online eingeben?*

3. *Gibt es Websiten/Online-Programme/Apps, die Du besuchst oder verwendest, wenn es Dir psychisch nicht so gut geht?*
    a. Falls ja: *Wie heissen sie?*
    b. Falls ja: *Wie hast Du sie gefunden?*
    c. *Was würde Dich überzeugen, dass diese Websiten/Online-Programme/Apps Jugendlichen helfen?*
    c. *Woran merkst Du bzw. würdest Du merken, dass sie Dir persönlich helfen?*
    d. *Woran erkennst Du bzw. würdest Du erkennen, dass diese Websiten/Online-Programme/Apps vertrauenswürdig sind?*

Barrieren und erleichternde Faktoren

Definition durch die interviewende Person:
Ich möchte Dir kurz etwas über unser Online-Programm erzählen, damit Du es Dir besser vorstellen kannst. Unser Online-Programm richtet sich an Jugendliche im Alter zwischen 12 und 19 Jahren, die aktuell unter leichten psychischen Problemen leiden. Es handelt sich dabei um ein Präventionsprogramm, da es darauf abzielt, die Entstehung von psychischen Problemen zu verhindern und die psychische Gesundheit zu fördern. Unter psychischer Gesundheit verstehen wir, dass Du Dich wohl fühlst, Deine Ziele erreichen kannst und mit Deinem Alltag, dem Zusammensein mit anderen Menschen und den Anforderungen in der Schule oder bei der Arbeit klarkommst.
Bei diesem Online-Programm wird es Informationen und Übungen zu verschiedenen Bereichen der psychischen Gesundheit geben (z.B. zum Umgang mit Stress oder einem geringen Selbstvertrauen). Die Teilnehmenden können sich online und anonym anmelden

und in ihrem eigenen Tempo die Inhalte am Computer, Tablet oder Handy machen. Da sie das Programm online selbstständig durchführen und nicht persönlichen Kontakt mit einem Therapeuten oder einer Therapeutin haben, bezeichnen wir das als ein Online-Selbsthilfeprogramm.
Für die Entwicklung eines solchen Programms stellen sich für uns einige Fragen, wie z.B. das Programm genau aussehen soll und welche Unterstützung sich die Teilnehmenden dabei wünschen würden. Jetzt wollen wir gemeinsam mit Dir und weiteren Jugendlichen herausfinden, wie wir unser Programm für Jugendliche möglichst ansprechend und interessant gestalten können.

4.
    a. *Welche Informationen über das Online-Selbsthilfeprogramm bräuchtest Du, um mitmachen zu wollen?*
    b. *Was könnten Gründe sein, dass Du nicht mitmachen möchtest?*
    c. *Was müsstest Du Deinen Mitschülerinnen und Mitschülern von dem Programm erzählen, damit sie mitmachen wollen?*

5. *Wann würdest Du ein solches Online-Selbsthilfeprogramm in Deinem Alltag nutzen?*

6. *Wie oft* (Logins pro Woche und Dauer pro Login) *und wie lange* (Gesamtdauer in Tagen oder Wochen) *würdest Du ein solches Online-Selbsthilfeprogramm gerne verwenden?*

7. *Gibt es etwas, was Du online gerne machst, wo Du dranbleiben musst, um Fortschritte zu erleben? Was motiviert Dich dranzubleiben? Was sind Gründe, dass Du vorzeitig abbrichst?*

8. *Könntest Du Dir vorstellen, 8-Wochen lang ein Online-Selbsthilfeprogramm zur Stärkung Deiner psychischen Gesundheit zu machen? Warum ja/warum nein? Was könnte Dich dazu motivieren, bis zum Schluss mitzumachen?*

Merkmale des Online-Programms

9.
    a. *Schätze bitte ein und begründe, wie wichtig Dir die folgenden Merkmale bei dem Online-Selbsthilfeprogramm wären (Skala 1-10: von «gar nicht wichtig» bis «extrem wichtig»).*
    b. *Wie könnte man diese Merkmale im Online-Programm am besten umsetzen?*

    - *Layout/Design*
    - *Informationstexte*
    - *Videos mit animierten Personen (wie in einem Cartoon)*
    - *Videos mit realen Jugendlichen*
    - *Videos mit Influencer:innen*
    - *Extra-Beispiele zum Anklicken (z.B. Beispiele von betroffenen Personen)*
    - *Erstellung eines eigenen Profils (z.B. wie auf Instagram)*
    - *Wahl eines Avatars (z.B. wie bei einem Computerspiel)*
    - *Austausch mit anderen betroffenen Jugendlichen (online)*

- *Austausch mit E-Coaches via Chat (d.h. durch extra dafür geschulte Fachpersonen)*
- *Austausch mit Fachpersonen (z.B. Psycholog:in) im persönlichen Kontakt (z.B. in einer Praxis)*
- *Einbezug Deiner Eltern*
- *Rückmeldung zu den erzielten Fortschritten*
- *Fortschrittsanzeige im Programm*
- *Belohnung bei erzielten Fortschritten*
- *Datenschutz*
- *Anonymität*
- *Selbstständig durchführbar*
- *Download/Nutzungsmöglichkeiten offline*
- *Aufgaben/Übungen, die selbstständig im Alltag gemacht werden*
- *Aufgaben/Übungen zur Selbstbeobachtung*
- *Anderes: ____________*

Personalisierung des Online-Programms

10. *Wie könnten wir das Online-Selbsthilfeprogramm möglichst gut an die einzelne Teilnehmerin bzw. den einzelnen Teilnehmer anpassen?*

11. *Das Online-Selbsthilfeprogramm richtet sich an Jugendliche im Alter zwischen 12-19 Jahren. Wie könnten wir das Programm so gestalten, so dass es für Jugendliche unterschiedlichen Alters geeignet ist?*

12. *Das Online-Programm richtet sich an Jugendliche jeglicher Geschlechtszugehörigkeit. Wie könnten wir das Programm so gestalten, dass es für Jugendliche jeglicher Geschlechtszugehörigkeit geeignet ist?*

Abschluss und weiteres Vorgehen

Herzlichen Dank fürs Mitmachen und Deine spannenden Antworten! Deine Antworten werden wir nun zusammen mit den Antworten der anderen Jugendlichen auswerten und dann nutzen, um unsere erste Version des Online-Selbsthilfeprogramms zu entwickeln. Auch dafür möchten wir wieder Jugendliche um ihre Einschätzung bitten. Dürften wir uns dafür erneut bei Dir melden, um Dich zu fragen, ob Du dann auch wieder mitmachen möchtest?

Nochmals vielen Dank fürs Mitmachen und alles Gute.
