# Supplementary material for: Adolescents’ Perspectives on Uptake, Engagement, and Implementation to Inform the Development of a Preventive Transdiagnostic Digital Mental Health Intervention: Qualitative Interview Study
Source: J Med Internet Res. 2026 Nov 2;28:e76494. doi: 10.2196/76494 (PMC13727026; doi:10.2196/76494)
Supplement: Supplementary file 2 [file jmir-v28-e76494-s003.pdf]

Multimedia Appendix 3. Extended Author Reflexivity Statement

The four master students who conducted the interviews were closer in age to the participants, which may have facilitated open discussions. At the same time, this proximity may have introduced unconscious biases, such as shared assumptions about digital engagement. Occasionally, participants referenced digital tools or ideas for DMHIs without further explanation, possibly assuming shared knowledge with the interviewers, which may have influenced how certain topics were explored in follow-up questions.

Between data collection and analysis, the research team gained further expertise in digital mental health and qualitative methods. This evolving knowledge likely informed the analytical process, as greater familiarity with the field may have led to more nuanced interpretations of the data. The delayed coding process also provided the benefit of increased analytical distance, potentially reducing immediate biases from the data collection phase.

The all-female composition of the research team may have influenced both data collection and interpretation, particularly regarding gendered experiences of mental health. During analysis, coders became aware that they tended to place significant weight on gender influences in adolescent experiences, possibly more than the participants themselves explicitly expressed. This reflexive awareness prompted a revisiting of the data to ensure that interpretations accurately reflected the participants' own priorities rather than the team's assumptions.
