# Supplementary material for: Adolescents’ Perspectives on Uptake, Engagement, and Implementation to Inform the Development of a Preventive Transdiagnostic Digital Mental Health Intervention: Qualitative Interview Study
Source: J Med Internet Res. 2026 Nov 2;28:e76494. doi: 10.2196/76494 (PMC13727026; doi:10.2196/76494)
Supplement: Supplementary file 3 [file jmir-v28-e76494-s004.pdf]

Multimedia Appendix 4. Codebook with main topics, subtopics, code descriptions and representative quotes

Codes and Subcodes	Code Description	Representative Quote
**Facilitating factors in the awareness phase of help-seeking**	Statements about what previously influenced and what is expected to influence adolescents' awareness of DMHIs to facilitate uptake and engagement with DMHIs	
Basic knowledge about DMHIs and their relevance for prevention	Statements about knowledge adolescents need to have about DMHIs and their relevance for prevention to enhance uptake or engagement	TN10(17):First, I feel like it should be about knowing that something like this exists and what it actually does. And also, if the idea is to intervene early and to have a preventive effect, I feel like it's definitely a question of how I would come across it, or how people can be encouraged to feel like, "Yes, this is actually helpful if I do it'," "It's worth doing this." It would definitely need to go hand in hand with raising awareness about the topic and also a certain normalization, like it's general knowledge that you need to have - how to take care of your mental health, just like you need to know how to put on a bandage.
Own previous experiences with digital (mental) health services	Statements about own experiences with digital (mental) health services that influence the future uptake or engagement with other DMHIs	TN37(15): What I found really annoying about them was that I once called, and I was stuck in the waiting queue because too many people were calling. That's kind of … yeah, when you need help or just need someone to talk to in that moment, and then you're stuck in a queue because everyone is busy, you just think, "Great, thanks a lot."
**Beliefs about DMHIs influencing uptake and engagement**	Statements on beliefs about potential benefits and barriers influencing uptake and engagement with DMHIs	
Improving knowledge about mental health	Statements showing that adolescents would uptake and engage with DMHIs because they have an interest in learning about (their) mental health and they would gain information and knowledge through their participation	TN7(21): Uhm, this might be simply my opinion but personally, I like to read up first and gather some information regardless of having to talk to someone already. Like, if I would want to know something [about mental health], if I could go on there and read about it.
Improving or maintaining personal mental health	Statements showing that adolescents would uptake and engage with DMHIs because they want to care for their mental health and protect them against mental health problems	TN16(15): Well, if it would personally help me, then [I would] definitely [use it] in those moments when I'm not feeling well, like when my mental health isn't good or whatever. […] Or maybe also as a preventive measure, maybe to avoid feeling mentally unwell in the future or build some kind of protection against that.

Providing entertainment and feedback	Statements showing that adolescents would uptake and engage with DMHIs if they provide entertainment and/or feedback	TN2(16): Well, the thing with levels is that when you somehow feel like, "level completed" or with a symbol or something, that really does a lot for me.
Beliefs about general helpfulness	General statements that adolescents' uptake and engagement depend on the expected helpfulness of the DMHI, which is not specifically related to mental health	TN41(17): [to make my friends want to use a DMHI] I would have to be able to tell them that it is really very helpful.
Critical beliefs about DMHIs and their usefulness for prevention	Statements in which adolescents express critical beliefs about self-help DMHIs and their usefulness for prevention	TN33(19): I am more inclined to think that it's of little use to me, but anyway, of course I don't rule it out completely and say 'no, I won't even try it, it's no use anyway' because maybe there will finally be a program that actually delivers results.
Expected stigmatization related to uptake and engagement of DMHIs	Statements about expected stigmatization if adolescents would uptake or engage with a DMHI	TN1(16): To me a name that describes it well so that one can find it [would be important for DMHI uptake]. However, that could be difficult because if it is called "mental health problems" and you have it on your phone […] and everyone sees it… I can imagine that for some this could make them feel really uncomfortable if someone takes a look at your phone, which happens quite often I think, and sees [the DMHI].
**Requirements for DMHI features**	Based on the assumption that adolescents have some experience with apps in general, some standards have evolved that they seem to perceive as "must haves" in any app	
Free of charge	Statements that DMHIs are expected to be free of charge	TN9(18): [Something not yet explicitly asked but important to me, is that it is free of charge] because if something costs money on the Internet, it's really discouraging.
State-of-the-art app	Statements that DMHIs should look, and be used like other state-of-the-art apps (including the appearance of the intervention and the technical aspects)	TN35(18; nb): I would use it on my phone because I always have it with me. And if it's a bit annoying to use on there, you know, like to navigate or if everything keeps shifting around and is just kind of awkward, I think with time it would start to really frustrate me.
**Preferences regarding presentation of DMHI content**	Adolescents' views and ideas on how the program content could or should be presented to appeal to them	
Content is easy to understand and follow	Statements about the way content is presented to make it easy to understand and follow	TN43(12): That you can do everything on your own and don't need help anywhere. That everything is clearly described and thoroughly explained so that you can understand everything. And yeah, that you don't really need

		help to figure something out, but that everything is relatively easy to understand.
Variety in delivery format	Statements that the program should include different delivery formats (e.g., text, video, podcast, etc.) to appeal to adolescents	TN19(17): […] if there's some variety. Like, switching between informational texts, maybe sometimes a video, or texts that other people have written. That it keeps changing a bit, so you can kind of look forward to the next time, but without knowing exactly what form it will take. But still knowing that something cool is coming that can help you. Something like that.
Inclusivity in content	Statements that stress the importance of an inclusive and diverse portrayal of content and youths (e.g., age, gender, skin color, weight, LGBTQIA+, religion)	TN20(16): It's really important that [the language] and everything is gender-inclusive, so that no one feels like they're not being addressed. And also, that it's not always the typical things - like, for example, if it's about relationship problems, there are definitely people dealing with that, but it shouldn't always be about a heterosexual couple in a closed, monogamous relationship. There should be diversity represented there as well.
Length and amount of content	Statements on individual perception of manageable duration or length or number of texts, exercises or overall intervention (NOT statements about the actual number of minutes, hours or logins per week)	TN35(18): [What I would need to know to use the DMHI?] Maybe how long an exercise takes? Like, if it lasts half an hour or whatever … I don't know, I think I'd be a bit like, "hmm." But if it's around 10 minutes a day or maybe 15 minutes, I think I'd be willing to spend that time for my mental health.
Personalization	Statements on personalization of content (tailored content based on user data)	TN33(19): I imagine that in the beginning, you fill out a questionnaire to assess where there are challenges. This could provide some guidance on what to focus on, while still allowing users to input their own areas for improvement. I would suggest that the program be somewhat personalized from the start.
Individualization	Statements on program parameters adolescents would like to individualize (users can choose from different options)	TN31(18): Well, I said I'm a fan of dark mode, but not everyone is, so it would be good if you could customize that or maybe choose a different design. Also, you should be able to set up your avatar.
**Ideas and wishes for adaptive, integrated and low threshold mental health services**	Statements about adolescents' ideas of how an ideal healthcare system should be organized	
Adaptive in intensity	Adolescents describe support services as being adaptive based on their needs or symptom severity (stepped care)	TN42(18): […] That you know: "okay, from these symptoms onward or whatever…" might be the point where one should seek professional help instead of relying solely on the program. But in general, the aim of the program is not seeking help from a person but to be able to solve [your

		problems] online on your own. So a note [about available services] would be important but not that they are a part of [the intervention].
Integrated and accessible	Adolescents describe support services as being integrated into DMHI, such as connecting with professionals through phone calls or face-to-face meetings	TN7(21): […] because if you realize - based on the information or maybe some tips provided there - something like, "If you’re feeling this way, it might be good to see a psychologist or psychiatrist," I would find it very helpful if that option were offered directly within the program. That way, you wouldn’t have to search for someone on your own.
Low threshold and continuous support	Adolescents describe that help should be easily available (especially, when they need it immediately) or that they will find what they need without any problems	TN29(18): Basically, I feel like it would help if the website guided you step by step. That you can just go on it without needing anything beforehand - except maybe thinking, “Hmm, I’m not feeling great, and maybe I just want to get some information and see: does it make sense to seek help, or is everything actually okay?” And then you kind of get guided through the process naturally, without already having to know exactly what’s wrong or thinking, “I have this and that issue, and I need help now.” Instead, you could just go on the website, even if you’re not sure, and it would guide you all the way.
**Discrepancies between expectations and implementation possibilities**	Statements that point to discrepancies between expectations and what may be possible to be implemented in mental health services	
Personalization without personal information	Statements indicating that adolescents are hesitant to share personal information but expect personalized feedback or support from the DMHI	TN38(18): [I don’t necessarily have ideas to implement data security.] That’s why I didn’t really like the idea of creating profiles, because they reveal a lot of information. [While] data protection is important, in case of an emergency, e-coaches should still be able to access basic data like an address or name if needed.
Permanent support with low personal engagement	Statements that indicate adolescents would expect immediate help to be available 24/7 and they would also prefer the help to be locally rooted but with only limited personal engagement	TN5(21): Well, in terms of login, I think it would be good if it were accessible at any time. Especially if you need help or some advice, or if you just want to get information, it should be easy to access directly when you need it.
Self-disclosure by others but not by themselves	Statements that indicate adolescents would appreciate it to read or hear from others who experience similar/same problems (and that reading/hearing this would be beneficial) but they would not want others to see or read what they are	TN18(21): I think it's important that [exchange with other adolescents] exists. But for me, it's always a bit uncomfortable to actually participate in the exchange. However, I do like reading when others share their experiences.

	experiencing due to stigma related to mental health and due to reluctance for self-disclosure	
**Trust**	Statements that are cutting across multiple analytic levels	
Recommendations from trusted sources	Statements that uptake or engagement would depend on recommendations from friends, family, school or (social) media	TN21(17): Well, in my school there are posters everywhere promoting […] some website or an app. I would probably try these first [if I ever needed help for my mental health].
Trustworthy provider	Statements about data security and the credibility/trustworthiness of the provider are essential	TN10(17): I feel like it should clearly state where it comes from, who is behind it and what their motivation is for creating it. To me, seeing that it's an intervention from a university and that there are studies behind it, that would definitely create a sense of trustworthiness. And I feel like it needs a clear statement like "About us" and a way for me to associate [the intervention] with something trustworthy and legitimate, or otherwise there should be some way to demonstrate why [the intervention] is trustworthy.
Testimonial reports	Statements about testimonials saying that the intervention is helpful or has benefits	TN31(18): Hm, I think the most important thing would be testimonials from others. And if they said it helped them, I would definitely give it a try as well.
Options for anonymous use	Statements about the need to offer options for an anonymous use of the DMHI	TN39(18): Additionally, that no personal information needs to be provided. Like, that you don't have to enter your name or, I don't know, your date of birth or anything. That these things remain optional.
**Support offered through a DMHI**	Statements stressing adolescents desire for support provided by the program such as that they feel supported by knowing they can access it at any time or from anywhere, when they need help	
Help is available and real	Statements that adolescents would want to know there was someone to help them soon, sometimes explicitly expressing it should be someone "real" (not AI) even when the help is accessed online	TN5(21): And if I knew that in an emergency, I could get help or whatever [on] there every evening for approximately 30 minutes it would provide me with a huge sense of reassurance in that moment.
Others share similar experiences	Statements that it would be reassuring to know that others have the same or similar experiences	TN19(17): What I also consider cool is if you can read stories from others about how they're doing. I think that helps so you don't feel as alone, or you don't think "it's just me".
Wanting to feel seen, understood, taken seriously	Statements on their desire to feel seen, listened to and taken seriously by the provider (including therapists / e-coaches) when they open up about their mental health issues	TN15(19): [I would need to know] if the person [on the other side] is genuinely interested in me. [And I would like] to feel like I am being taken seriously for real and to feel like the questions and content [in the DMHI] is applicable.
