# Supplementary material for: Adolescents’ Perspectives on Uptake, Engagement, and Implementation to Inform the Development of a Preventive Transdiagnostic Digital Mental Health Intervention: Qualitative Interview Study
Source: J Med Internet Res. 2026 Nov 2;28:e76494. doi: 10.2196/76494 (PMC13727026; doi:10.2196/76494)
Supplement: Supplementary file 4 [file jmir-v28-e76494-s005.pdf]

Multimedia Appendix 4. Detailed Implementation Suggestions Related to their Impact on Uptake or Engagement

UPTAKE → ENGAGEMENT

Recommendations from friends, family and school:
- Provide information on details what was helpful
- Provide information on source of the recommendation to facilitate relatability

Anonymity
- Optional data provision (whenever possible)
- Using nicknames or usernames not real names

Requested information
- Registration process
- Time investment
- Outcome expectancies
- Provider qualifications
- Required personal information
- Handling of personal data
- Content is well-founded
- Content is up-to-date
- Clearly state intervention goals

Fostering trust
- "About us" section (including duty of confidentiality, credentials, age, own mental health experiences)
- Providing references for the content of the intervention
- Transparency about financing
- Placing data security information prominently

Stigma
- Raising awareness about mental health in adolescents and parents
- Learning how to talk about mental health problems with peers and family
- Chat possibilities with professionals instead of calling or face-to-face appointments

Testimonials
- Report on helpfulness of the intervention
- Stories of others about their experiences
- Stories of others about their coping strategies
- Possibilites for peer exchange

Appealing design
- Color scheme
- Simple and sleek design
- Modern and minimalistic
- Clear structure

Design features
- Like button
- Saving responses and strategies to personal profile
- Download options

Low-threshold, integrated, adaptive mental health care service
- Booking appointments with professionals via DMHI
- Sending e-mails to professionals via DMHI
- E-coaches for mild symptoms and mental health care professionals for more severe symptoms
- Emergency button connecting users with professionals
- No registration process
- No waiting times
- Intervention reaching out when symptoms deteriorate
- Intervention directly connecting users with similar experiences
- Intervention suggesting relevant mental health care services nearby

Individualization
- Customization and depiction of profiles/avatars (gender, sexuality, body type, skin color, age, hairstyle, clothing style)
- Customizable interface (light/dark mode, font, color scheme)
- Privacy options (private or public profile, anonymity options, push notification preferences)
- Exchange options (peers, family system, professionals, e-coaches)
- Content (level of difficulty, problem focus, filter option for duration of exercises, option for more in-depth information)

Gamification/Rewards
- Levels to be unlocked
- Token systems
- Smiley faces
- Encouraging messages from the DMHI

Mental health goals
- Progress bar
- Personalized feedback on progress
- Self-checks on progress
- Goal overview

Delivery format
- Variety of formats (e.g., podcasts, audios, short texts, videos (informational, testimonials, lived experiences), interviews (adolescents and experts), communication examples, interactivity, exercises
- Language (gender-neutral, providing audio versions of text)

Personalization
- Content based on questionnaire responses
- Content based on primary concerns
- Content adapts dynamically based on user input

Peer support
- Digital self-help group
- Chat with others who have similar experiences
- Interactive comment sections (e.g., below exercises)

IMPLEMENTATION
