# Supplementary material for: Adolescents’ Perspectives on Uptake, Engagement, and Implementation to Inform the Development of a Preventive Transdiagnostic Digital Mental Health Intervention: Qualitative Interview Study
Source: J Med Internet Res. 2026 Nov 2;28:e76494. doi: 10.2196/76494 (PMC13727026; doi:10.2196/76494)
Supplement: Supplementary file 5 [file jmir-v28-e76494-s002.docx]

## Multimedia Appendix 2. Importance Ratings of Predefined Intervention Features

Mean value of feature ratings and standard deviation

Predefined features

Note. ^a^n=47, ^b^n=40.
